# Benefits of Minimal Access Surgery in Elderly Patients with Pelvic Cancer

**DOI:** 10.3390/cancers8010012

**Published:** 2016-01-12

**Authors:** Vincent Lavoué, Walter Gotlieb

**Affiliations:** 1 Service de chirurgie gynécologique, Centre Hospitalo-Universitaire de Rennes, Hôpital Sud, 16 Bd de Bulgarie, 35000 Rennes, France; vincent.lavoue@gmail.com; 2 Inserm, ER440-OSS, CRLCC Eugène Marquis, Avenue Bataille Flandre-Dunkerque, 35000 Rennes, France; 3 Division of Gynecologic Oncology, Segal Cancer Center, Jewish General Hospital, McGill University, Montreal, QC H3T 1E2, Canada

**Keywords:** robotic surgery, minimal invasive surgery, cancer, elderly

## Abstract

An increasing proportion of patients requiring treatment for malignancy are elderly, which has created new challenges for oncologic surgeons. Aging is associated with an increasing prevalence of frailty and comorbidities that may affect the outcome of surgical procedures. By decreasing complications and shortening length of hospital stay without affecting oncologic safety, surgery performed using the robot, rather than traditional laparotomy, improves the chances of a better outcome in our growing elderly populations. In addition to age, surgeons should take into account factors, such as frailty and comorbidities that correlate with outcome.

## 1. Introduction

Life expectancy continues to rise and doubled during the last century. Over the past 20 years alone, the population older than age 65 years doubled, and the population older than age 85 quadrupled [1]. Over the next 20 years, the “baby boom” generation will require more and more health care as they advance into older age [2]. This has created a new challenge and placed a large burden on health care resources in oncology as the increasing proportion of patients requiring treatment for malignancy are elderly [3], and the cohort of people over the age of sixty-five accounts for almost two-thirds of new cancer cases and three-fourths of cancer related deaths [2]. Elderly are less likely to be offered standard cancer treatments because of the lack of clear guidelines, and the intuitive concerns of clinicians and family members about the ability of elderly to tolerate surgery. They often present with more advanced disease and higher-risk histology [4,5,6,7], and have historically been under-represented in clinical trials [1,8,9,10,11,12,13,14], so sufficient evidence is not available for the development of standardized treatment guidelines for this growing patient population, especially concerning the surgical options. They are frequently considered for surgical resection, although complication rates, mortality, length of hospital stay, and intensive care unit admissions increase with patient age [8]. Recent data show the feasibility of surgical treatment in elderly in several types of tumors, such as sarcomas [15,16], gynecologic cancer [6,17], urologic cancer [18], colorectal cancer [19], or pulmonary surgery [20].

If surgery is determined to be the appropriate treatment modality, including in the elderly, it remains challenging. Aging is associated with an increasing prevalence of frailty, comorbidities, decline of functional reserve, and progressive restriction in personal and social resources, which result in a greater vulnerability, functional decline, institutionalization, and falls [21]. Normal physiologic changes occur in major organs and may affect the response to surgical procedures [8,22,23]. In this context, minimally invasive surgery, or more precisely minimally access surgery (MIS) could be particularly valuable, because MIS has been shown to be less physiologically disruptive than open surgery, especially in the elderly [20,24]. In addition, multiple comparative studies have demonstrated that MIS is associated with shorter hospital stays and convalescence, less morbidity, and achieves similar oncologic outcomes as open surgery [18,19,25,26,27]. Furthermore, robot-assisted laparoscopic surgery, considered as a natural evolution of laparoscopy, is becoming widely accepted [28] because it allows more patients to benefit from MIS. Retrospective cohort studies and meta-analyses showed that robotics was a safe and efficient surgical approach for several types of pelvic surgery, improving outcome compared to open surgery [6,29,30,31]. Some concerns were raised when using robotics in the elderly. Once the patient is docked to the robot, the steep Trendelenburg position cannot be reversed without undocking, and the respiratory and cardiovascular systems might be adversely affected by the prolonged Trendelenburg position and this might compromise the potential advantages of robotic surgery in the elderly population [6,32,33,34]. In view of the limited data regarding its value in the elderly population, this review aims to better define elderly population, and to analyze the potential benefits and risks of MIS compared with open surgery.

## 2. Elderly: Age *vs.* Frailty?

Most studies that deal with elderly patients and surgery used an arbitrary cut-off of 65- or 70- years of age [35,36,37,38]. Age represents an independent risk factor for morbidity and mortality [4,39,40], which associated with some surgical procedures will lead to unacceptably high risks of postoperative complications. However, unjustly denying elderly patients surgical procedures solely on the basis of age or inaccurate assessment tools may deny them potentially life saving, or quality of life improving surgical procedures. Defining elderly patients based on functional status might be more accurate than age to define risks associated with surgery [41]. Current surgical risk estimations are imprecise because the traditional risk indices are overly subjective and do not account for physiologic reserve. Although most physicians intuitively recognize frailty, the lack of standardized assessments introduces much subjectivity into surgical decision-making. The inaccuracy, imprecision and inconsistency amongst the doctors in assessing patient life-expectancy has significant implications for managing patients [42]. Of note, elderly patients who survive the first year after surgery have the same cancer-related survival as younger patients and should undergo optimal therapeutic approaches, including optimal surgery [43].

Medical geriatrics aims to more accurately define patient′s health state and introduced the concept of frailty. Frailty is defined as a discrete syndrome of decreased physiologic reserve that limits a patient′s ability to respond to stressors and predisposes patients to adverse outcomes [44,45]. Frailty identifies adults that are at increased risk for falls, hospitalizations, and other adverse outcomes [21]. Most incorporate physical, cognitive, social, and biochemical components [46,47] and the frailty criteria described by Fried *et al.* were capable of providing accurate evaluations in prospective surgical studies [21,48], especially in patients with gynecologic cancers [49]. Frailty assessment as a preoperative risk estimation tool allowed identification of patients susceptible to postoperative complications, institutionalization, increased length of stay and mortality after surgery [41,48,50,51]. Several studies showed that frailty assessment outperforms the traditional risk assessments of the ASA (American Society of Anaesthesiologists), ECOG (Eastern Cooperative Oncology Group) performance status and CCI (Charlson Comorbidity Index) [48,52,53,54].

Although MIS decreases overall post-operative complication rates compared to open surgery, severe complications remained similar [6]. A better understanding of frailty might help guide the extent of surgery and the optimal surgical approach. If oncology practice requires TNM or FIGO staging system assessment, onco-geriatric practice also requires frailty assessment in order to tailor surgical (either MIS or open surgery) and other therapeutic approaches [55].

## 3. Benefits of MIS in Elderly Patients Compared to Open Surgery

From a patient′s perspective the value of a surgery can be represented by an equation where the outcome is divided by the invasiveness of the procedure. The less invasive the procedure for a similar outcome the higher the value for the patient. This is particularly true in the frail patient whose recovery can be severely compromised by a more invasive procedure. MIS in oncology is characterized by minimal incisions and pneumo-peritoneum to allow enough abdominal space to perform complex dissections while avoiding long scars required for open surgery. The benefits or MIS are now well established for different kind of cancers, including colorectal [56,57], urologic [25,27,38], pancreatic [26], and gynecologic cancers [6,32,58,59]. Theses include decreased blood loss, quicker return of bowel function, shorter length of hospitalization stay, less postoperative pain, fewer wound infections, lower incidence of postoperative pneumonia, lower incidence of postoperative cardiac complications, and quicker convalescence [25,26,60,61,62]. The only downside appears to be longer operative times, but data on elderly populations in surgical oncology remain scarce. Although endometrial cancer is the most common gynecologic malignancy in the western world, with increasing incidence, parallel to the aging demographics [63,64,65], only six published studies specifically address surgery in the elderly (Table 1). The data show improved benefits of MIS compared to open surgery in the elderly, similar to their younger counterparts [30,34,56,60]. For patients with colon cancer, there was no statistically significant difference between younger and older patients in incidence of postoperative death, anastomotic leak, or postoperative ileus [60]. In a large retrospective review, using the American College of Surgeons National Surgical Quality Improvement Program (NSQIP), outcomes data demonstrated an overall protective effect of laparoscopy for major complications (intensive care unit admission and mortality), but that a higher frailty score in the laparoscopy cohort remained correlated with major complications but that MIS avoids major complications in part of the elderly patients when compared with open surgery, but not in all elderly patients, notably not in the more frail patients [66]. These findings are in accordance with data of our group that showed that robotics decreases minor complications (grade I and II Clavien Dindo complications [67]) but not major complications (grade III and IV Clavien Dindo complications) in the most frail elderly population over the age of 80 when compared to open surgery [6], suggesting that a subgroup of elderly patients are still subject to major complications, that is inherently associated with the anaesthesia and surgical insult that cannot be avoided by MIS. Identification of this subgroup of elderly patients before surgery by assessing frailty, might help avoid surgery altogether and consider a conservative therapeutic approach.

## 4. Benefits of Robotic Surgery Compared to Laparoscopy

The Da Vinci Surgical System**^®^** could be viewed as the natural evolution of laparoscopy. Reports comparing robotics to laparotomy have demonstrated reduced operative blood loss, lower incidence of postoperative complications, and faster recovery resulting in shorter hospital stay [58,59,68,69,70,71,72], with comparable recurrence rates and survival [6,58,70]. Although little data is available comparing robotics to laparoscopy, some authors reported benefits of robotics over standard laparoscopy in the treatment of endometrial cancer, with significantly less blood loss (74.5 mL *vs.* 145.8 mL), decreased operative time (191 min *vs.* 213 min) and higher node counts (32 nodes *vs.* 23 nodes) as a surrogate for better surgery [59,73]. An additional clinically significant benefit of robotic surgery is the lower conversion rate compared to standard laparoscopy, mainly in complex surgical procedures such as nephrectomy [31], and in patients with high body MISs index (obese and morbidly obese women) [74,75]. This reduced conversion rate could be of particular value in elderly patients, who were found to have a 30% increase of conversion for each decade of age in a large randomized trial comparing laparotomy to laparoscopy for the surgical management of endometrial cancer [76].

**Table 1:** Studies about surgical staging in elderly with endometrial cancer.

Study	Number of Patients	Age (Mean, Years)	Surgical Approach	Operation Time (mn)	Blood Loss (mL)	Nodal Yield (n)	Hospital Stay (Days)	Transfusion Rate	Overall Complications Rate
Scribner *et al.* (2001) [32]	45	76	Open	148	336	29	5.8	19%	69%
	67	75	Laparoscopy	236	298	29	3.0	2%	28%
Susini *et al.* (2005) [35]	43	74	Open	115	400	NR	8	NR	7% *
Lachance *et al.* (2006) [61]	151	>65 **	Open	176	384	NR	8	NR	40%
Moore *et al.* (2007) [33]	42 (staged patients)	84	Open or Laparoscopy	NR	NR	NR	6.7	14%	21% ***
Lowe *et al.* (2010) [62]	27	84	Robotic	192	50	16	1	0%	0%
Lavoue *et al.* (2014) [6]	113	78	Robotic	244	75	10	3	3%	21%
	50	77	Open	217	334	9	8	10%	66%

NR: Not Reported. * only major complications; ** mean age was not reported; *** only reported transfusion and readmission rate.

Finally, the main advantage of robotic surgery compared to laparoscopy is to increase MIS access to patients. Although elderly patients may particularly benefit from MIS, the adoption of standard laparoscopy has been slow [61,77,78]. Similar to many centers, only 17% of patients with endometrial cancer benefitted from MIS using laparoscopy in our academic centre. Since the introduction of robotics in our hospital in 2007, the number of patients undergoing MIS for endometrial cancer has increased within two years to reach over 95% [6,58] and has remained stable since. This increase resulted from a full commitment to evaluate the role and value of robotic surgery, by offering this surgical approach to each operable patient unless the cancer could not be extracted intact (or in a 15 cm diameter endobag) via the vagina. The ability of the robotic computer interface to facilitate intuitive movements similar to conventional surgery, facilitated the learning curve [59,79], contrasting with the more complex approach required by strait stick laparoscopy. Conventional laparoscopy can safely be performed by most surgeons in less complex surgeries, but requires highly skilled and talented laparoscopic surgeons in high-risk patients, such as those with cancer, the obese, and the elderly. The advent of robotic surgery had decreased the use of laparotomy, providing increased benefits in the more frail elderly population, who are at higher risk for postoperative complications.

## 5. Limits of Robotic Surgery

The physiologic effects of pneumo-peritoneum, steep Trendelenburg, and more prolonged surgeries, may lead to postoperative problems unique to MIS. In addition, once the patient is docked to the robot, the Trendelenburg position cannot be reversed without undocking, and the respiratory and cardiovascular systems might be adversely affected by the Trendelenburg position and compromise the potential advantages of robotic surgery in the elderly. A case of cerebral oedema following robotic surgery was reported [80] and prolonged Trendelenburg increases the potential risk of blindness in patients suffering from moderate or high-pressure glaucoma [81]. Despite these case reports, most data support the safety of robotic surgery in the general population [70] and in the elderly [6,82].

A limitation of the three first generations ofrobotic platforms consisted of the static position once docked, that did not allow a significant change in direction of the robot towards a new surgical field without undocking, placement of some new trocars, and re-docking [83,84]. The newest Da Vinci robot, named Xi**^®^,** has resolved this limitation and allows for multiple quadrant access without the need to undock.

Another important consideration has been cost and resource allocation. High acquisition and maintenance costs have placed the robotic platforms beyond the reasonable reach of many institutions. Nevertheless, advantages of robotic surgery such as shorter length of stay and reduced morbidity, improve the cost effectiveness in Western type health care systems. For example, Bell *et al.* [85] showed that the average cost for the surgical treatment of an endometrial cancer was highest for laparotomy, followed by robotic and standard laparoscopy. The total average cost for hysterectomy with staging completed via laparotomy was $12,943.60, for standard laparoscopy $7569.80, and for robotic assistance $8212.00. The difference in cost between laparotomy and robotic was significant *p* = 0.0001, while there was no statistically significant difference in cost between laparoscopy and robotic (*p* = 0.06) [85]. Similar results were found in endometrial cancer when comparing robotic surgery to the mix of laparotomy and laparoscopy [58].

## 6. Oncologic Safety of (Robotic) MIS

The oncologic safety of robotic surgery remains difficult to determine in elderly. Indeed, data are limited on the long-term outcomes in elderly patients and whether surgical technique has an effect on survival [60]. Nevertheless, decrease mortality in the first year after surgery is crucial for elderly (and thus the less morbidity surgery) because some data showed that elderly patients who survive the first year after surgery have the same cancer-related survival as younger patients [43]. Robotic could challenge perioperative care and 1-year outcomes after surgery.

The oncologic safety of robotic surgery seems similar with open surgery or laparoscopic surgery according results of Bogess′s study with five-year follow-up post robotic surgery in endometrial cancer context [70]. After two years of follow-up, data suggest robotic surgery in elderly patients is safe from an oncology point of view in terms of comparable rates of progression-free survival [6].

As conclusion, robotic surgery seems do not compromise oncologic safety and could increase outcome of oncologic disease because robotic surgery decrease morbidity and mortality of surgery, especially in frail elderly patients.

## 7. Considerations for the Future

Robotics represents the natural evolution of minimal access surgery, with the addition of a computer interface between the surgeon and the patient. This computer at present only allows improved magnified vision and dexterity of the surgeon, but this interface is expected to evolve in the future with integration of pre-operative or even intra-operative imaging in the surgeons visual field, to digitally analyze variations in temperature of tissues, color, movement such as pulsations, and integrate immunofluorescence to delineate target organs. These improvements in the information provided to the surgeon during surgery could be compared to the information provided to pilots during flight, including feedback during surgery and data analysis following surgery.

## 8. Conclusions

Minimal access surgery (MIS) benefits elderly patients by significantly decreasing peri-operative complications, improving quality of life, and accelerating their return to normal activities. At present, a subgroup of the frailest elderly still remains at increased risk of major complications that interfere with outcome results of surgical treatments. Overall, surgeons should be aware of the major advantages offered by MIS and work towards improving preoperative frailty assessments that will help tailor the right surgery for the right subgroup of elderly patients.
